# Developmental trajectories of visual temporal integration and segregation in children with and without developmental dyslexia

**DOI:** 10.1111/bjdp.70010

**Published:** 2025-08-21

**Authors:** Giuseppe Di Dona, Alessia Santoni, David Melcher, Luca Ronconi, Laura Franchin

**Affiliations:** ^1^ School of Psychology Vita‐Salute San Raffaele University Milan Italy; ^2^ Department of Psychology and Cognitive Science University of Trento Rovereto Italy; ^3^ Psychology Program, Division of Science New York University Abu Dhabi Abu Dhabi United Arab Emirates; ^4^ Center for Brain and Health, NYUAD Research Institute New York University Abu Dhabi Abu Dhabi United Arab Emirates

**Keywords:** developmental disability, developmental dyslexia, developmental trajectories, integration, learning disorder, segregation, visual perception

## Abstract

In the present study, 43 Italian school‐age children (age range = 7–14 years, 16 females) with (*N* = 19) and without DD (*N* = 24) were presented with pairs of visual displays separated by varying interstimulus intervals and performed either a temporal integration or segregation task despite an identical visual input. Children with DD had lower accuracy and slower RTs for longer temporal intervals. Additionally, efficiency (combined accuracy and speed trade‐off) increased as a function of age only in the DD group, most markedly for the integration condition. Results suggest that visual temporal processing deficits in DD may depend on short‐term/working memory liability as well as the existence of possibly differentiated developmental trajectories for integration and segregation abilities.

**Key points**

- Children with developmental dyslexia show differentiated developmental trajectories for visual temporal integration and segregation.
- Such trajectories possibly reflect the development of the M‐D stream, alpha oscillations and visuospatial working memory.
- Accuracy at text reading is correlated to temporal integration skills in typically developing children but not in children with developmental dyslexia.

## INTRODUCTION

Developmental Dyslexia (DD) is a neurodevelopmental condition characterized by difficulties with accurate and/or fluent word recognition and by poor spelling and decoding abilities (Peterson & Pennington, 2015). These primary manifestations may lead to life‐long secondary consequences impacting emotional, social and occupational experience (Beetham & Okhai, 2017). Given the long‐term impact of DD alongside its high prevalence, estimated to range between 5 and 20% in the general population (Wagner et al., 2020), there is a continuous drive to unravel its primary manifestations.

Various hypotheses have been suggested to explain its diverse symptoms, from issues in phonological processing to problems in sensory and attentional processing. Phonological abilities, crucial for understanding written and spoken language, play a key role in reading acquisition (Melby‐Lervåg et al., 2012). However, phonological impairments alone cannot fully characterise the multifaceted nature of dyslexia (Pennington, 2006; Snowling et al., 2020). Together with phonological abilities, reading also requires the accurate sequencing of visual input into graphemes and word forms and ultimately integrates visual and auditory features over time (Stein, 2019).

A key challenge for the ‘reading brain’ lies in its need to break down ongoing sensory information into coherent segments, like visual elements and spoken units. On the one hand, swiftly processing and reacting to shifts in sensory inputs is vital for recognising changes and directing actions. On the other hand, the capacity to merge information over time is essential for gathering evidence and making accurate choices based on more precise inputs. Therefore, the act of processing sensory information in time necessitates a careful equilibrium between integration and segregation. Perception across the senses is grounded in temporal processing, which shapes conscious experience on the basis of intrinsic neuronal timescales, essentially determining whether separate physical events are perceived as a unified experience or not (Golesorkhi et al., 2021; Wolff et al., 2022). It has been suggested that the key brain structures guiding spatiotemporal integration and segregation lie along the magnocellular‐dorsal (M‐D) visual stream (Maunsell & Newsome, 1987). The M‐D stream is a fast visual sub‐pathway which computes the spatiotemporal coordinates of visual events by sequentially selecting specific elements in the visual field, for further processing by the slower parvocellular‐ventral (P‐V) stream, which is primarily involved in perceptual binding and object recognition (Bullier, 2001, 2002; Kveraga et al., 2007; Nassi et al., 2006; Saalmann et al., 2007). Given its functions, the M‐D stream gives key contributions to motion perception and scene analysis (Rafal, 2024), attentional allocation (Martínez et al., 2013), visuomotor coordination (Cooper & O'Sullivan, 2016), saccades preparation and planning (Curtis & Connolly, 2008) and ultimately to reading whose efficiency depends from all of the previous. Any dysfunction in the M‐D system—anywhere from the lateral geniculate nucleus (LGN) to the superior parietal cortex—may result in slower and less accurate pre‐saccadic attentional shifts (Giraldo‐Chica et al., 2015; Graboi & Lisman, 2003; Jaœkowski & Rusiak, 2005; Müller‐Axt et al., 2017; Vidyasagar, 1999, 2019; Vidyasagar & Pammer, 2010), difficulties in correctly integrating or segregating letters and words in the visual field (Gori et al., 2016; Gori & Facoetti, 2015) and increased susceptibility to visual crowding (Bertoni et al., 2019; Martelli et al., 2009; Zorzi et al., 2012).

Anomalies in such temporal processing mechanisms might play a role in DD. In particular, given that both pure linguistic and perceptual deficits have been repeatedly found in reading disabilities, one important question is whether there is a core sensory processing component that could underlie both issues (Goswami et al., 2014). The ‘classic’ theory of rapid temporal processing deficits, proposed by Tallal in Tallal, 1980, highlights how auditory processing issues, particularly with fast sequences of sounds, may underlie phonological impairments in DD (Tallal, 1980). As an analogue framework developed to explain visual temporal problems, the magnocellular‐dorsal (M‐D) theory of DD suggests that dyslexia depends on deficits in processing fast‐changing visual information and controlling eye movements due to abnormalities in the magnocellular pathway of the visual system. Such M‐D pathway impairments have been linked to difficulties in contrast sensitivity, motion perception and oculomotor adjustments during reading (Di Dona et al., 2024; Di Dona & Ronconi, 2023; Stein, 2019; Stein & Walsh, 1997; Turri et al., 2023). Early assessment of the M‐D stream sensitivity, related to these pathways, can predict literacy skills (Gori et al., 2016; Kevan & Pammer, 2009), suggesting that M‐D deficits might affect various levels of sensory and attentional processing (Gori et al., 2016; Hari & Renvall, 2001).

In the attempt to link temporal processing deficits to attentional deficits, the ‘Sluggish Attentional Shifting’ (SAS) theory further elaborates on how dyslexia might involve slower shifts in attention across rapidly presented sequences, leading to issues in processing and segregating visual and auditory information quickly (Hari & Renvall, 2001). This difficulty is also tied to challenges in directing attention quickly enough to relevant graphical elements during reading, implying a broader problem with attentional mechanisms in visual tasks (Franceschini et al., 2012; Krause, 2015; Vidyasagar & Pammer, 2010). Temporal processing has been found to be abnormal in individuals with DD relative to controls, both in auditory (Goswami, 2002; Hornickel & Kraus, 2013; Tallal, 1980) and visual tasks (Boets et al., 2011; Galaburda & Livingstone, 1993; Gori et al., 2016; Gori & Facoetti, 2015; Kevan & Pammer, 2009; Menghini et al., 2010; Tallal, 2004), as well as in spatiotemporal mechanisms of attention (Bosse et al., 2007; Facoetti et al., 2010; Roach & Hogben, 2007; Visser et al., 2004). Some works have reported that children with DD performed worse in tasks requiring rapid visual processing (Di Lollo et al., 1983; Facoetti et al., 2008; Stanley & Hall, 1973), leading to the corollary proposed by Di Lollo et al. (1983) that the ‘dyslexic visual system may take an unusually long period to recover from the aftereffects of neural activity evoked by an inducing stimulus’ (p. 923). However, other studies reported no such deficits (Arnett & Di Lollo, 1979; Fisher & Frankfurter, 1977).

To demonstrate the role of temporal processing as a key factor in reading (dis)abilities, it is critical to control for other confounding variables that might be involved in the tasks. In tasks probing temporal processing, it might be difficult to take apart specific temporal processing differences from more general performance factors, such as sustained attention, processing speed, response bias, motivation or motor‐/response‐related problems. Recent studies employing a segregation–integration (‘SegInt’) task in adult participants with DD have explored visual temporal processing deficits with both psychophysics and event‐related potentials (ERPs), showing that adults with DD struggle more with segregating rapidly presented visual stimuli, unlike their peers without DD (Ronconi et al., 2020; Santoni et al., 2024). In the SegInt task, a variant of the missing element task (Di Lollo, 1980), participants see two displays of stimuli separated by a varying interstimulus interval (ISI). Each of the two displays contains seven full annuli, placed in random locations within an invisible 4 × 4 grid, and an ‘odd element’ with a half annulus, such that the two half annuli completed each other across displays in the same position. Based on task instructions, participants had to find the position of the single odd to perform a segregation task, which thus requires separating the two displays over time to avoid confusing it with the other full annuli. Conversely, in the integration condition, the task was to find the position of the single location that was left empty, which can be achieved by integrating the displays over time. Thus, the manipulation of task instruction in the SegInt task allows researchers to disambiguate temporal integration and temporal segregation processes, while maintaining visual stimulation identical across conditions. Previous evidence has extensively tested the SegInt task to assess temporal integration and segregation performance in adults (Ronconi et al., 2018, 2020, 2022; Santoni et al., 2024; Sharp et al., 2019; Wutz et al., 2016, 2018) and in children, with ages ranging from 5 to 7 years (Freschl et al., 2019) and even in 2‐year‐old toddlers (Freschl et al., 2021).

In typically developing 5‐ to 7‐year‐old children, overall performance on the SegInt task grows as a function of age, possibly reflecting a developmental trajectory of processing speed during visual search (Freschl et al., 2019). It is very important to consider that visuospatial processes depend on the development of visual working memory (WM), whose capacity is directly related to the maturation of frontal networks (Buss et al., 2018). Such abilities are impaired in DD as suggested by atypical patterns in neurophysiological indexes of temporal attention and working memory ( Kranczioch et al., 2005; Lotfi et al., 2022; Ronconi et al., 2016; Santoni et al., 2024). Therefore, it is important to consider that the developmental trajectories of integration and segregation abilities are influenced by the concurrent development of visual WM. In particular, WM may play a role in temporal segregation, as it requires maintaining 2 separate displays in memory, while integration may require WM to actively merge the two displays, especially with long temporal intervals.

While some studies described the developmental trajectories of rapid temporal processing of typically developing children (Freschl et al., 2019), the developmental trajectories for temporal integration and segregation of children with DD have never been reported. In the present study, we used the SegInt task to explore the trajectories of temporal processing development in school‐age children with or without DD. Specifically, we investigated whether reading impairments are linked to an overall anomalous ability to quickly process sensory inputs or to a specific temporal segregation deficit as recently suggested by studies using the SegInt task in adults with DD (Ronconi et al., 2020; Santoni et al., 2024) and by seminal works with other tasks in children with DD (Di Lollo et al., 1983; Facoetti et al., 2008; Laasonen et al., 2000).

Based on the segregation deficit found in adults with DD with the SegInt task (Santoni et al., 2024), we expect to find such deficits also in children with DD in terms of accuracy. Considering this segregation deficit in adult age, we expect a slower performance growth (which may also reach a plateau) as a function of age with respect to typically developing children. Instead, considering the absence of integration deficits at an adult age (Ronconi et al., 2020; Santoni et al., 2024), we might expect a comparable developmental trajectory between children with and without DD. Ultimately, to fully characterise the processing dynamics of temporal segregation and integration, we will also capture RTs and compute inverse efficiency to explore possible speed–accuracy trade‐offs.

## METHODS

### Participants

A total of 43 Italian children were recruited for the study (16 females, 27 males; mean age = 11.12, age range 7–14 years).^1^ A total of 19 participants have been diagnosed with DD by a certified neuropsychologist, while 24 neurotypical control participants presented no history of reading difficulties. The majority of participants with DD presented at least a comorbidity with another learning disorder (see Table S1), with the most common comorbidity being dysorthography (present in 14 participants). The study was approved by the ethical committee of the University of Trento, and parents gave written informed consent for the participation (Table 1).

**TABLE 1:** Demographic information and neuropsychological testing differences between the two groups.

Measure	Group		Statistics
	Controls (*n* = 24)	DD (*n* = 19)	Two‐sample *t*‐tests
	Mean (SD)	Mean (SD)	
Age	10.90 (1.65)	11.30 (1.59)	*t* (39.34) = −0.78, *p* = 0.43
YOE	5.92 (1.74)	6.11 (1.37)	*t* (41) = −0.39, *p* = 0.69
Raven	0.56 (1.28)	0.16 (1.08)	*t* (40.81) = 1.11, *p* = 0.27
Text reading speed	0.29 (0.83)	−1.78 (1.31)	*t* (29.06) = 5.98, *p* < .001
Text reading accuracy	0.23 (0.79)	−2.59 (1.96)	*t* (22.66) = 5.92, *p* < .001
Words reading speed	0.01 (0.80)	−3.81 (2.22)	*t* (21.71) = 7.23, *p* < .001
Words reading accuracy	−0.44 (1.48)	−2.60 (2.49)	*t* (27.70) = 3.33, *p* = .002
Pseudowords reading speed	0.26 (0.98)	−2.96 (2.47)	*t* (22.52) = 5.37, *p* < .001
Pseudowords reading accuracy	0.15 (0.81)	−2.02 (1.79)	*t* (23.87) = 4.92, *p* < .001

### Neurocognitive assessment

Participants were tested with Raven's Coloured Progressive Matrices (children up to 11 years old) or Standard Progressive Matrices (for older children) (Raven & Raven, 2003) to test general intelligence regardless of reading and linguistic skills. Reading ability was tested by asking children to read aloud a short text extracted from the MT‐3 reading test battery for primary and lower secondary school (Cornoldi & Carretti, 2016) and lists of words and pseudowords extracted from the DDE‐2 battery (Sartori et al., 2013). Reading ability was assessed in terms of accuracy (Z‐scored number of errors) and speed (Z‐scored syllables per second). Both reading tests are commonly used for the diagnosis of DD and have been validated on large samples of Italian children.

### SegInt task

**FIGURE 1:**
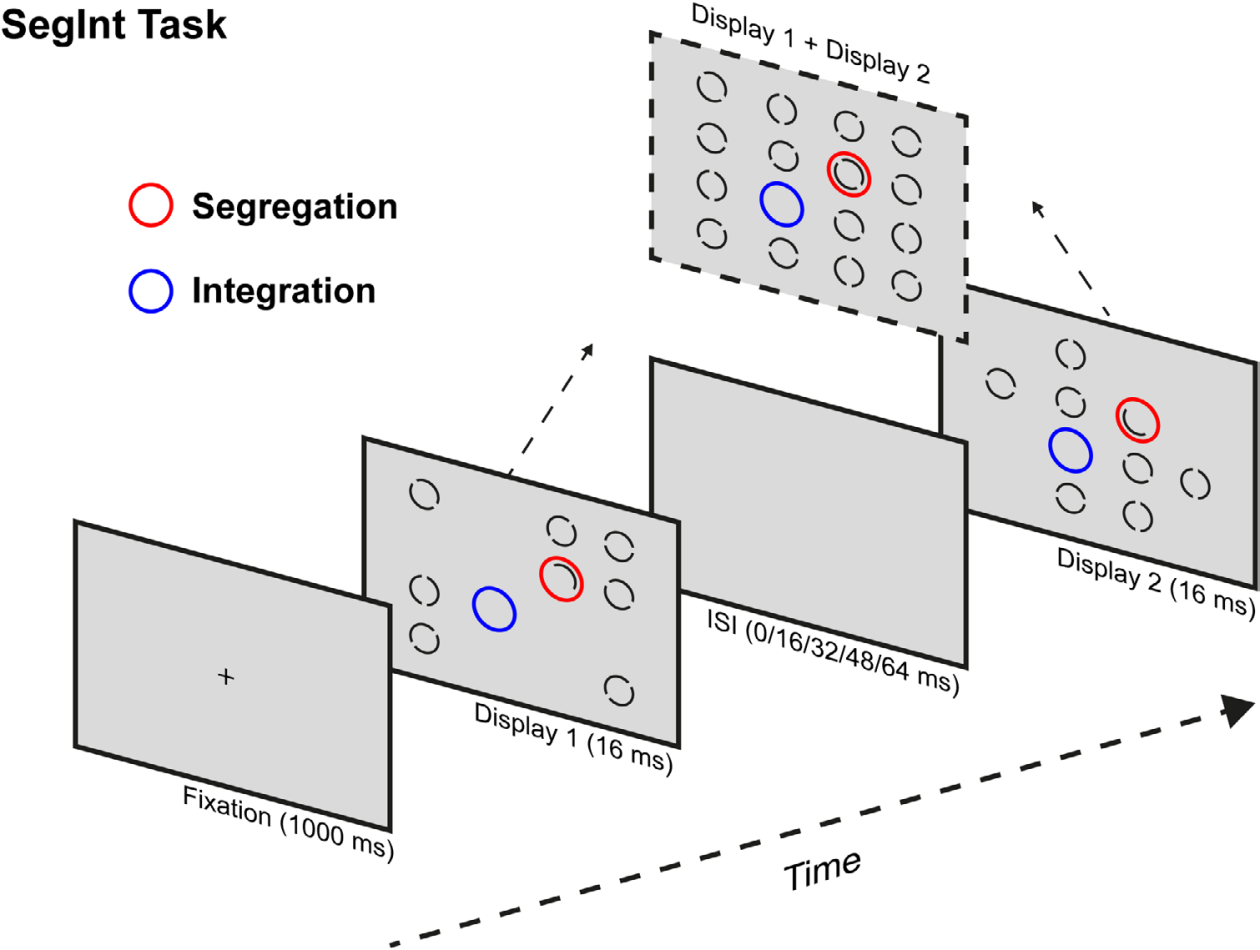
Schematic representation of the SegInt task and the stimuli employed for the present study.

Visual temporal processing was assessed by means of the SegInt task, a modified version of the missing element task (Di Lollo, 1980). The task was implemented using E‐prime version 3.0 software (Psychology Software Tools, Pittsburgh, PA, 2016) and administered on a portable 13.3‐inch laptop (32.5 × 22.5 cm) with a resolution of 1920 × 1080 pixels at a refresh rate of 60 Hz. Stimuli were presented on a light grey background and consisted of two target displays presented for 16 ms each and separated by a blank screen with varying ISI (0, 16, 32, 48, 64 ms). Each target display consisted of an invisible 4 × 4 quadratic grid centred on the screen; 7 of the 16 possible positions on the grid were randomly filled with a full annulus (separated by a central gap with orientation varying between 0°, 45°, 90° or 135°) and 1 position with an odd stimulus, a half annulus. Annuli were presented at a size of ~0.85°, interspaced by ~0.14° and with a line width of ~0.13°. The invisible quadratic grid containing the annuli subtended ~6.12°. Each trial started with a fixation cross (1000 ms), followed by the presentation of the two target displays. Between displays of the same trial, only one random position on the grid was left empty (considering the two overlapped displays), while another random position contained an odd stimulus, a half annulus with mirrored orientation between displays. Following this, a response prompt appeared in the form of a quadratic grid; children had to click on the location where they thought that the target stimulus appeared using a computer mouse (Figure 1).

### Procedure

After the neurocognitive assessment, children were presented with the SegInt task. Prior to task execution, children were familiarised with the task with a short practice of integration and segregation blocks. In integration blocks, participants were instructed to pay attention to the one location left empty among target displays, which was possible only by temporally binding the two displays. In segregation blocks, children were asked to pay attention to the odd element (the half annulus with mirrored orientation among displays). Noticing the odd element was only possible if the two displays were not integrated; otherwise, it would have been perceived as a full annulus. This experimental design allows to isolate temporal integration and segregation processes by changing task instructions while keeping visual stimulation constant. The experimental session consisted of 4 alternating blocks of integration and segregation trials (2 blocks per Condition), each containing 30 trials for a total of 120 trials. The whole experimental session lasted approximately 40–60 min in total, including the neurocognitive assessment.

### Statistical analyses

Participants' accuracy of single‐trial data in the SegInt task was analysed via a generalised linear mixed‐effects model (GLMM) with a logit link function using the package lme4 in R (Bates et al., 2015). In the model, the predictors Condition (Segregation, Integration) and Group (Control, Developmental Dyslexia) were inputted as categorical factors, while the predictors ISI and Age were inputted as continuous covariates only after being scaled. Note that when continuous covariates are added, the effects are estimated over the mean value of the covariates. The model formula included all the fixed factors described above as well as all their possible interactions. Participants' ID was included as a random intercept.

Participants' RTs (log‐transformed) of single trials where a correct response was given were analysed via a linear mixed‐effects model (LMM) using the package lmerTest in R (Kuznetsova et al., 2017). In the model, the predictors Condition (Segregation, Integration) and Group (Control, Developmental Dyslexia) were inputted as categorical factors, while the predictors ISI and Age were inputted as continuous covariates only after being scaled. The model formula included all the fixed factors described above as well as all their possible interactions. Participants' number was included as a random intercept.

Participants' inverse efficiency index was computed by dividing the Mean RTs of correct trials by the mean accuracy for every cell of the experimental design, considering the factors Condition (Segregation, Integration) and Group (Control, Developmental Dyslexia). Then, inverse efficiency scores were analysed via a linear mixed‐effects model (LMM) using the package lmerTest in R (Kuznetsova et al., 2017) using all the factors of the experimental design as well as ISI and Age (scaled). The model formula included all the fixed factors described above as well as all their possible interactions. Participants' number was included as a random intercept.

To evaluate all the fixed effects and interactions, chi‐square tests were run on each of the models via the ‘Anova’ function of the package car in R (Fox & Weisberg, 2019). Post‐hoc comparisons were implemented via the emmeans R package (Lenth et al., 2018) on the estimated marginal means (EMMs) of each model. When analysing complex interactions involving multiple continuous predictors in post‐hoc analyses, such continuous predictors were also refactored as categorical, while analysing the slopes of the remaining continuous predictor. This means that the slopes of, for example, continuous predictor 1 were estimated at levels (e.g., A and B) of the continuous predictor 2. This applies to the continuous covariate Age, which was refactored as categorical with two levels (younger: 9.60 y.o.; Older = 12.60 y.o.), corresponding to the mean ± 1SD of the sample's age distribution. A similar approach was taken for the continuous predictor ISI which was sometimes refactored as categorical (Short: 16 ms; Long: 64 ms) by selecting the shortest and longest non‐zero ISIs. While studying the slopes as indexes of the developmental trajectories might not highlight differences in the ‘speed’ of such trajectories, refactoring the continuous covariates and comparing model predictions between two points of the continua might still reveal differentiated outcomes stemming from not clearly differentiated trajectories. When computing multiple comparisons, *p*‐values were corrected using the FDR correction (Benjamini & Hochberg, 1995).

Lastly, a correlation analysis was run on neuropsychological testing data and behavioural data from the SegInt task; first, separately within each group and then collapsing groups together. Scores from neuropsychological tests were z‐transformed considering their respective standardised conversion tables, while behavioural data were z‐transformed considering the mean and standard deviation of the whole sample. All correlations were controlled for the influence of Age. *p*‐values were corrected using FDR correction for multiple comparisons (Benjamini & Hochberg, 1995).

## RESULTS

### Accuracy

**FIGURE 2:**
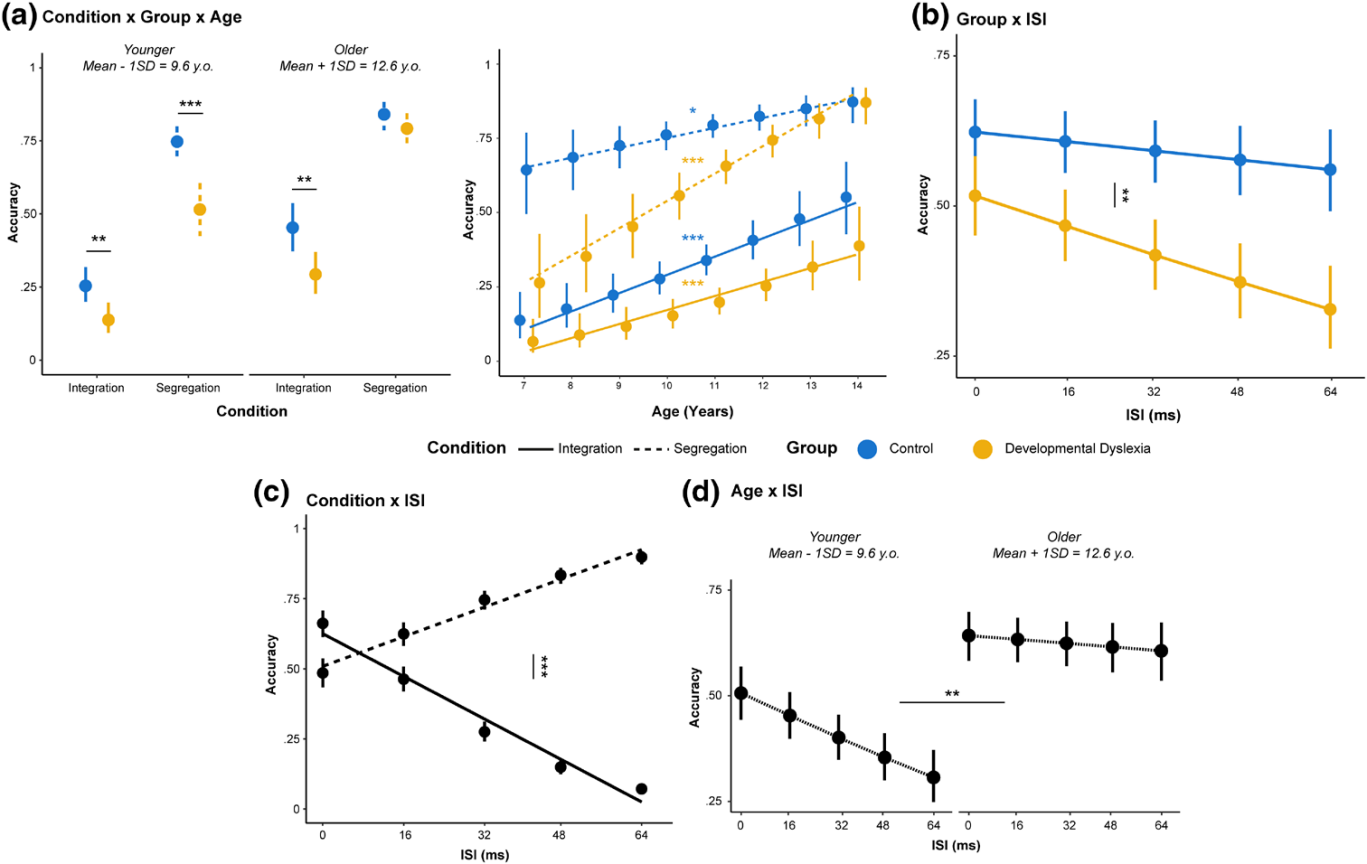
Interaction effects from the accuracy GLMM. (a) Interaction effects between Condition, Group and Age. In the leftmost panel, points and vertical lines indicate EMMs of Accuracy with 95% confidence intervals for Control (blue) and Developmental Dyslexia (yellow) in the Integration (solid line) and Segregation (dashed line) for Younger (9.6 y.o.) and Older (12.6 y.o.) participants. In the rightmost panel, the EMMs and the slopes are represented for the same interaction. Asterisks indicate the level of significance (*p < .05, **p < .01, ***p < .001). (b) Interaction effect between Group and ISI. Points and vertical lines indicate EMMs of Accuracy with 95% confidence intervals connected by the linear fit line for Control (blue) and Developmental Dyslexia (yellow). Asterisks refer to the post‐hoc comparison between the slopes of the groups. (c) Interaction effect between Condition and ISI. Points and vertical lines indicate EMMs of Accuracy with 95% confidence intervals connected by the linear fit line in the Integration (solid line) and Segregation (dashed line). Asterisks refer to the post‐hoc comparison between the slopes of the conditions. (d) Interaction effect between Age and ISI. Points and vertical lines indicate EMMs of Accuracy with 95% confidence intervals connected by the linear fit line for Younger (9.6 y.o.) and Older (12.6 y.o.) participants. Asterisks refer to the post‐hoc comparison between the slopes of the age groups (D).

The analysis of accuracy data showed a significant interaction between Condition, Group, and Age χ^2^(1) = 6.14, *p* = .013 (see Figure 2a). Post‐hoc tests revealed that younger Control participants showed higher accuracy in the integration (odds ratio = 2.43, SE = 0.58, *p* = .007) and in the segregation condition (odds ratio = 2.78, SE = 0.66, *p* < .001) with respect to younger DD participants while Older control participants only showed higher accuracy in the Integration condition (odds ratio = 2.00, SE = 0.49, *p* = .007) with respect to Older DD participants and not in the Segregation condition (Odds Ratio = 1.39, SE = 0.20). Post‐hoc tests performed on the Age slope showed that accuracy grew significantly higher in function of age for all groups and conditions but with no difference between them (Integration Control: Slope = 3.28, SE = 0.85, z‐ratio = 3.81, *p* < .001; Segregation Control: Slope = 2.13, SE = 0.84, z‐ratio = 2.52, *p* = .011; Integration DD: Slope = 3.52, SE = 1.03, z‐ratio = 3.39, *p* < .001; Segregation DD: Slope = 4.70, SE = 0.95, z‐ratio = 4.94, *p* < .001).

A significant interaction was also found between Group and ISI *χ* ^2^(1) = 5.46, *p* = .02 showing that the accuracy diminished faster for growing ISI in DD participants (Slope = −0.48, SE = 0.1) with respect to Control (Slope = −0.16, SE = 0.09; Slope difference = 0.32, z‐ratio = 2.36, SE = 0.14, *p* = .018) (see Figure 2b). A significant interaction between Condition and ISI *χ* ^2^(1) = 606.37, *p* < .001 revealed that Accuracy grew higher for growing ISI in the Segregation condition (Slope = 1.35, SE = 0.10) while it showed the opposite trend in the Integration condition (Slope = −2.00, SE = 0.10; Slope difference = −3.35, z‐ratio = 24.20, SE = 0.13, *p* < .001) (see Figure 2c). Furthermore, an interaction effect was found between ISI and Age *χ* ^2^(1) = 10.21, *p* = .001 (see Figure 2d). Post‐hoc tests revealed that accuracy diminished faster for growing ISI in younger participants (Slope = −0.54, SE = 0.10) with respect to older ones (Slope = −0.11, SE = 0.09; Slope difference = −0.43, z‐ratio = −3.81, *p* = .002). Post‐hoc tests performed on the Age slope showed that accuracy grew significantly faster with longer ISIs (Slope = 4.69, SE = 0.76) with respect to lower ISIs (Slope = 2.76, SE = 0.59; z‐ratio = −3.81, *p* = .002) in function of Age. The main effects of Condition (*p* < .001), Group (*p* < .001), ISI (*p* < .001) and Age (*p* < .001) were also statistically significant.

### Reaction times (RTs)

**FIGURE 3:**
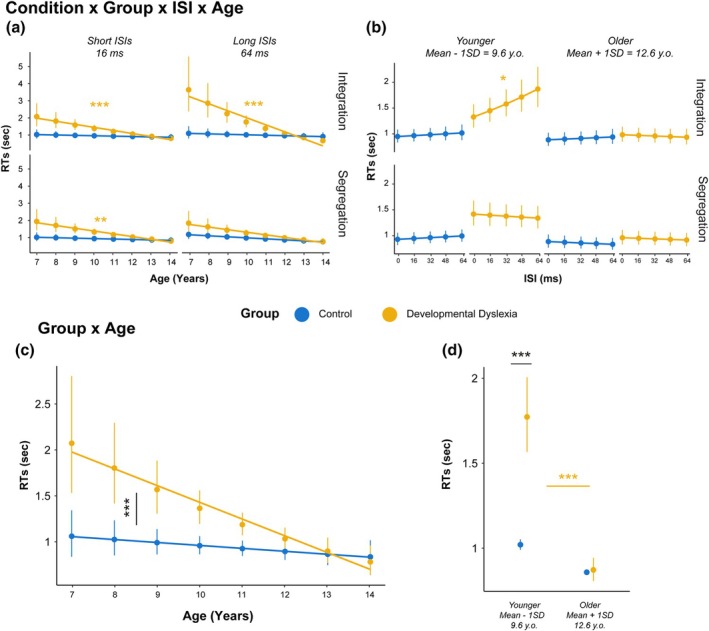
Interaction effects from the RTs LMM. (a) Interaction effect between Condition, Group, ISI and Age. Points and vertical lines indicate EMMs of RTs of correct trials with 95% confidence intervals connected by the slope of the effect of Age for Control (blue) and Developmental Dyslexia (yellow) in the Integration and Segregation conditions for short (16 ms) and long (64 ms) ISIs. Horizontal lines indicate the compared EMMs of post‐hoc comparisons: Black lines indicate between‐group comparisons, while coloured lines indicate within‐group comparisons. Asterisks indicate the level of significance (*p < .05, **p < .01, ***p < .001). (b) Interaction effect between Condition, Group, ISI and Age. Points and vertical lines indicate EMMs of RTs of correct trials with 95% confidence intervals connected by the slope of the effect of ISI for Control (blue) and Developmental Dyslexia (yellow) in the Integration and Segregation conditions for younger (9.6 y.o.) and older (12.6 y.o.) participants. (c) Interaction effect between Group and Age. Points and vertical lines indicate EMMs of RTs of correct trials with 95% confidence intervals connected by the slope of the effect of Age for Control (blue) and Developmental Dyslexia (yellow). (d) Interaction effect between Group and Age. Points and vertical lines indicate EMMs of RTs of correct trials with 95% confidence intervals for Control (blue) and Developmental Dyslexia (yellow) for younger (9.6 y.o.) and older (12.6 y.o.) participants.

The analysis of RTs showed an interaction between Condition, Group, ISI and Age χ (1) = 8.91, *p* = .002. Post‐hoc tests revealed that only younger DD participants in the Integration condition showed an increase in RTs for growing ISIs (Slope = 0.23, SE = 0.06, *t*‐ratio = 3.59, *p* = .041; Figure 3b). Additional tests also showed that RTs significantly decreased in function of Age only in DD participants for the segregation condition with short (Slope = −1.42, SE = 0.39, *t*‐ratio = −3.60, *p* = .002) and long ISIs (Slope = −1.39, SE = 0.40, *t*‐ratio = −3.42, *p* = .002) and also for the integration condition with short (Slope = −1.51, SE = 0.39, *t*‐ratio = −3.81, *p* = .001) and long ISIs (Slope = −2.71, SE = 0.50, *t*‐ratio = −5.33, *p* < .001; Figure 3a).

The simpler interaction effect between Group and Age χ (1) = 4.74, *p* = .029 showed longer RTs for younger DD participants (EMM = 1481, SE = 115) with respect to younger control participants (EMM = 974, SE = 58; *t*‐ratio = −4.26, *p* < .001; Figure 3d) but also compared to older DD participants (EMM = 947, SE = 66; *t*‐ratio = 4.32, *p* < .001). When analysing the slopes, DD participants showed a steeper reduction of RTs across age (Slope = −1.64, SE = 0.38) with respect to control participants (Slope = −0.25, SE = 0.32; *t*‐ratio = 2.58, *p* = .013; Figure 3c).

The effects of Condition (*p* = .010), Group (*p* = .001), Age (*p* < .001), as well as the interaction effects between Condition and ISI (*p* = .021), ISI and Age (*p* = .021) were also statistically significant.

### Inverse efficiency

**FIGURE 4:**
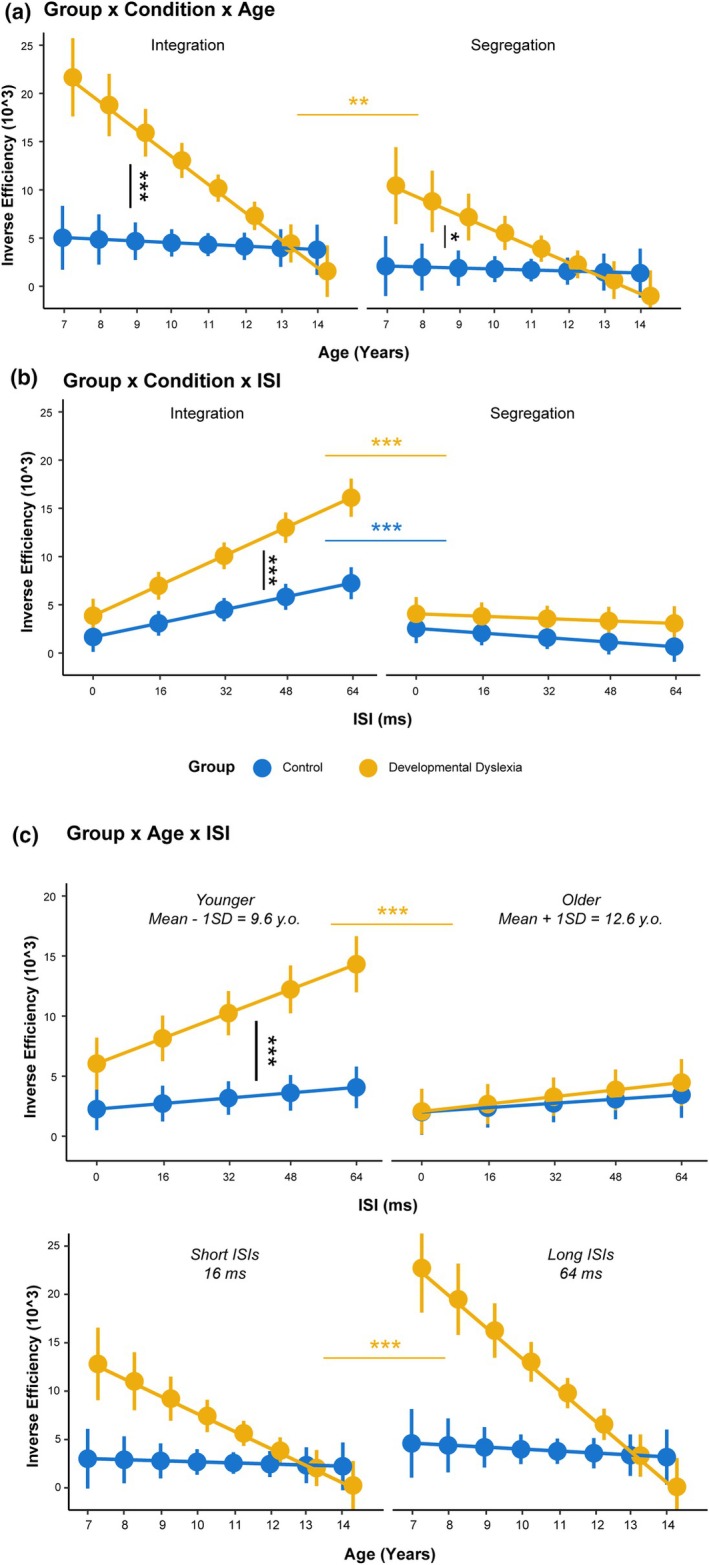
Interaction effects from the inverse efficiency LMM. (a) Interaction effect between Group, Condition and Age. Points and vertical lines indicate EMMs of Inverse Efficiency with 95% confidence intervals for Control (blue) and Developmental Dyslexia (yellow) in the Integration (solid line) and Segregation (dashed line) connected by the slope. Horizontal lines indicate the compared EMMs of post‐hoc comparisons: Black lines indicate between‐group comparisons while coloured lines indicate within‐group comparisons. Asterisks indicate the level of significance (*p < .05, **p < .01, ***p < .001). (b) Interaction effect between Group, Condition, ISI. Points and vertical lines indicate EMMs of Inverse Efficiency with 95% confidence intervals connected by the slope line for Control (blue) and Developmental Dyslexia (yellow), in the Integration (solid line) and Segregation (dashed line). (c) Interaction effect between Group, Age and ISI. The upper panel shows points and vertical lines indicate EMMs of Inverse Efficiency with 95% confidence intervals connected by the slope line for Control (blue) and Developmental Dyslexia (yellow), in Younger (9.6 y.o.) and Older participants (12.6 y.o.). Points and vertical lines in the lower plot indicate EMMs of Inverse Efficiency with 95% confidence intervals connected by the slope line for Control (blue) and Developmental Dyslexia (yellow), for short (16 ms) and long (64 ms) ISIs.

The analysis of the Inverse Efficiency revealed an interaction effect between Group, Condition and Age *χ* ^2^(1) = 5.49, *p* = .019. Post‐hoc tests revealed that Inverse Efficiency reduced faster with increasing age in DD participants (Slope = −32,214, SE = 4977) with respect to Control participants (Slope = −1998, SE = 4409; Slope difference = 30,216, SE = 6649, *t*‐ratio = 4.54, *p* < .001) in the Integration condition. The same pattern was observed for the Segregation condition as a larger slope was computed for DD participants (Slope = −18,334, SE = 4940) with respect to Control participants (Slope = −1162, SE = 4189; Slope difference = 17,173, SE = 6477, *t*‐ratio = 2.65, *p* = .014). Additionally, the reduction of Inverse Efficiency over increasing Age was faster for Integration with respect to Segregation only in DD participants (Slope difference = −13,880, SE = 4207, *t*‐ratio = −3.29, *p* = .002; Figure 4a). An interaction effect was also found between Group, Condition and ISI *χ* ^2^(1) = 5.92, *p* = .015. Post‐hoc tests showed that in the Integration condition, Inverse Efficiency grew larger in a faster way as ISI increased in DD participants (Slope = 7520, SE = 783) with respect to Control participants (Slope = 3427, SE = 660; Slope difference = −4093, SE = 1024, *t*‐ratio = −3.96, *p* < .001). No differences between the groups were found in the Segregation condition (Slope difference = −554, SE = 946, *t*‐ratio = −0.58, *p* = .55). Additionally, DD participants showed a larger slope in the Integration condition with respect to the Segregation condition (Slope = −609, SE = 709; Slope difference = 8130, SE = 1056, *t*‐ratio = 7.69, *p* < .001). The same pattern was found for Control participants (Slope = −1163, SE = 627; Slope difference = 4591, SE = 909, *t*‐ratio = 5.04, *p* < .001; Figure 4b). An interaction effect was found between Group, Age and ISI *χ* ^2^(1) = 5.35, *p* = .020. Post‐hoc test revealed that Inverse Efficiency increased faster for growing ISIs in younger DD participants (Slope = 5411, SE = 820) with respect to younger Control participants (Slope = 1249, SE = 659; Slope difference = −4163, SE = 1052, *t*‐ratio = −3.95, *p* < .001). Additionally, younger DD participants showed a steeper slope with respect to older DD participants (Slope = 1658, SE = 665; Slope difference = 3754, SE = 1046, *t*‐ratio = 3.58, *p* < .001). DD participants (Short: Slope = −20,373, SE = 4656; Long: Slope = −37,148, SE = 5652) also showed a steeper reduction of inverse efficiency in function of Age with respect to Control participants (Short: Slope = −1287, SE = 4148; High: Slope = −2287, SE = 4773) for short (*t*‐ratio = 3.06, *p* = .004) and long ISI (*t*‐ratio = 4.71, *p* < .001). Also, while DD participants showed steeper Age slopes for long vs. short ISIs (*t*‐ratio = 3.85, *p* < .001), control participants did not (Figure 4c).

The effects of Condition (*p* < .001), Group (*p* < .001), ISI (*p* < .001), Age (*p* < .001), as well as the interaction effects between Condition and Group (*p* < .001), Condition and ISI (*p* < .001), Group and ISI (*p* = .003), Condition and Age (*p* = .022), Group and Age (*p* < .001), ISI and Age (*p* = .013) were also statistically significant.

### Correlations

**FIGURE 5:**
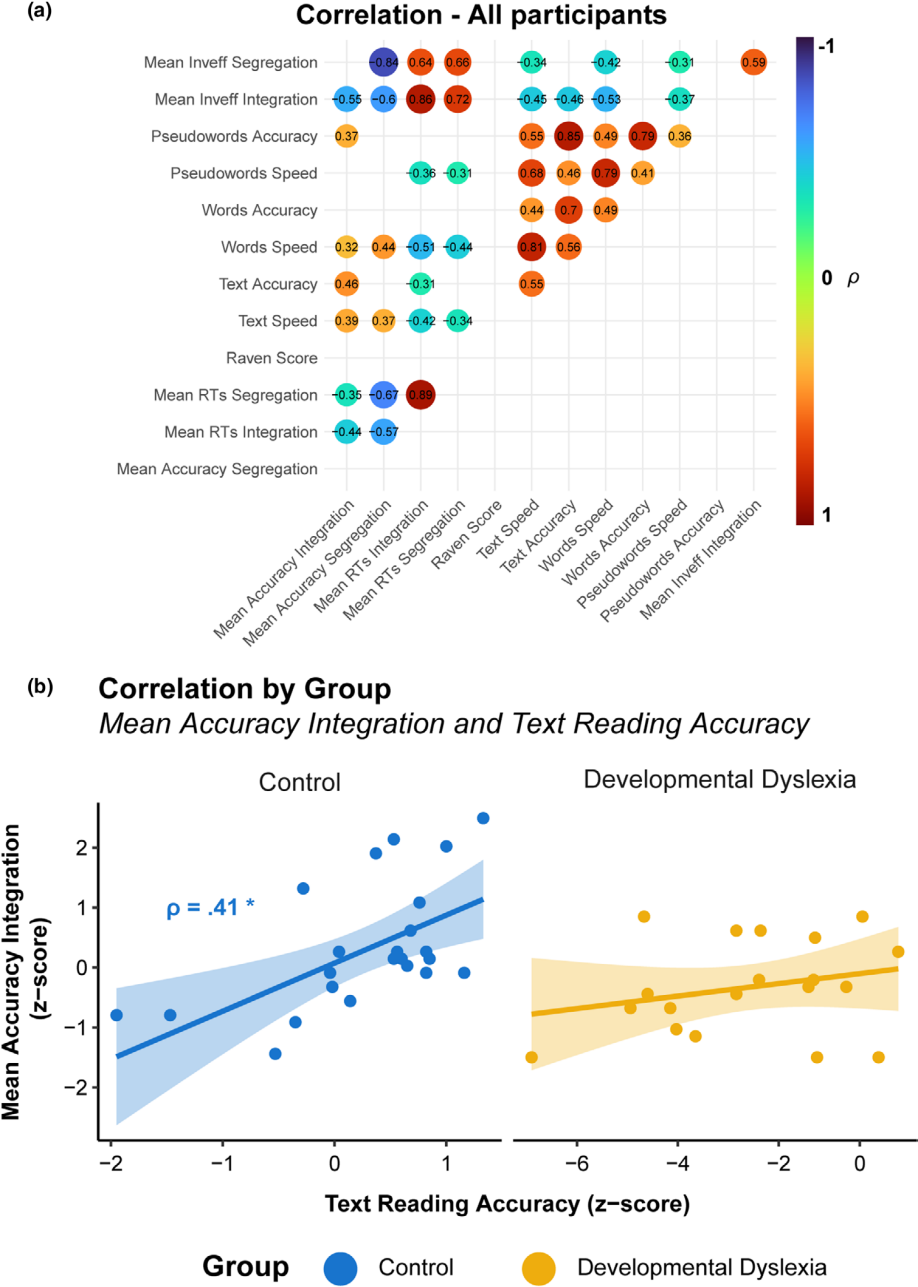
Correlation between neuropsychological and behavioural data. (a) A correlogram of the correlation tests controlled for age operated on the whole sample. Red dots indicate positive correlations while blue dots indicate negative correlations. In each dot, *ρ* is indicated in numbers (b) Scatterplot of the correlation between Mean Accuracy in the Integration condition (z‐transformed) from the SegInt task and the Text Reading Accuracy (z‐transformed) from neuropsychological testing. Dots indicate mean values for each individual participant with the linear fit line. Asterisks indicate the level of significance of the correlation (*p < .05, **p < .01, ***p < .001).

In the Developmental Dyslexia Group, no correlations between neuropsychological and behavioural data were found. In the Control group, a positive correlation between the Text Reading Accuracy and the Mean Accuracy in the Integration condition (*ρ* = .41, *p* = .05; see Figure 5).

When collapsing all groups, positive correlations between the Mean Accuracy in the Integration condition and the Text Reading Accuracy (*ρ* = .46, *p* = .002) and Speed (*ρ* = .39 *p* = .040). Positive correlations were also found between the Mean Accuracy in the Segregation condition and Words Reading Speed (*ρ* = .44, *p* = .003) and Text Reading Speed (*ρ* = .37, *p* = .015). Negative correlations were found between Mean RTs in the Integration condition and Pseudowords Reading Speed (*ρ* = −.36, *p* = .018), Words Reading Speed (*ρ* = −.51, *p* < .001), and Text Reading Speed (*ρ* = −.42, *p* = .005). A positive correlation was also found with Text Reading Accuracy (*ρ* = .31, *p* = 045). Negative correlations were also found between Mean RTs in the Segregation condition and Words Reading Speed (*ρ* = −.44, *p* = .003), Text Reading Speed (*ρ* = −.34, *p* = .027) and Pseudowords Reading Speed (*ρ* = −.31, *p* = .046). Similarly, negative correlations were also found between the Mean Inverse Efficiency (which takes into account both RT and accuracy) in the Integration (Text Speed *ρ* = −.45, *p* = .002; Text Accuracy *ρ* = −.46, *p* = .002; Words Speed *ρ* = −.53, *p* < .001; Pseudowords Speed *ρ* = −.37, *p* = .015) and Segregation Condition (Text Speed *ρ* = −.34, *p* = .029, Words Speed *ρ* = −.42, *p* = .005, Pseudowords Speed *ρ* = −.30, *p* = .047). Furthermore, several correlations were found between the different variables of the neuropsychological data, and the same applied to behavioural data, both within each group and within the whole sample of participants (see Supporting Information).

## DISCUSSION

The SegInt task, here tested on school‐age children, has been confirmed as a valid option for measuring visual integration and segregation mechanisms, in agreement with previous studies in younger children (Freschl et al., 2019, 2021). Indeed, we could dissociate temporal segregation from integration mechanisms only by changing task instructions in both neurotypical children and children with DD. More specifically, all children showed an increase in accuracy and a decrease in RTs as the ISI increased in segregation blocks, while the opposite emerged in integration blocks, where children showed a decrease in accuracy and longer RTs for increasing ISIs (Figure 2c). The results also included an expected general effect of age on performance, that is, older children have higher accuracy and shorter RTs. Furthermore, younger children in both groups showed a decrease in accuracy as the ISI increased. Thus, regardless of the task‐specific demands, processing stimuli separated by longer time intervals was more challenging for younger children.

With respect to group differences, we expected a segregation deficit in children with DD, potentially emerging with a different developmental trajectory from the one of neurotypical children. In parallel, we expected comparable trajectories for integration abilities. In terms of accuracy, we found only partially differentiable trajectories for integration and segregation performance between children with and without DD. While we showed no differentiation of the improvement slopes across the two groups and conditions (Figure 2a, 2nd panel), when testing for differences in the predictions of our model in younger (9.6 y.o) and older children (12.6 y.o.), we found that while integration deficits persisted at both ages, segregation deficits were apparently absent at 12.6 y.o. (Figure 3a, 1st panel). However, when looking at RTs, distinct trajectories emerged in terms of processing time, showing marked age‐related improvements in DD children for integration trials (Figure 3a). Integration difficulties across the ages are also highlighted when testing for differences in the predictions of our model, as at 9.6 y.o. DD children showed longer processing times (Figure 3b). By jointly analysing accuracy and RTs, we also observed distinct patterns in inverse efficiency, with DD children showing faster improvement as a function of age, especially for the integration condition (Figure 4a). Together, these results reveal a clear impairment of spatiotemporal integration abilities in children with DD, affecting processing times and efficiency. This impairment is characterised by a developmental trajectory that diverges not only from the one of typical children but also from the one pertaining to segregation abilities. In contrast, we found segregation impairments were less pronounced, with minimal or no differentiation in their related developmental trajectories.

Our hypotheses were drawn by previous studies investigating temporal processing with the SegInt task in adults with DD (Ronconi et al., 2020; Santoni et al., 2024, 2025) showing a specific segregation deficit, accompanied by spared integration skills. This pattern was accompanied by a generally reduced N1 ERP component in DD participants, as well as a task‐induced modulation of the P3 component, showing greater amplitude for temporal segregation only in neurotypical adults (Santoni et al., 2024). According to the authors, a reduced N1 component for both integration and segregation trials possibly suggests a weaker deployment of attentional resources that would lead to weaker stimulus representations in visual WM, as shown by the absence of a P3 modulation. These electrophysiological signatures support visuo‐attentional theories of DD, such as the SAS (Hari & Renvall, 2001) framework and the magnocellular theory (Stein & Walsh, 1997; Stein, 2019), suggesting that perceptual and attentional deficits might trigger cascade effects on WM and, ultimately, reading ability. This interpretation is supported by further evidence showing that adults with DD also struggle to tune the core oscillatory rhythms of visual sampling. Specifically, a top‐down modulation of alpha oscillations, modulating visual temporal sampling depending on task demands, was observed only in typical readers (Santoni et al., 2025), in line with the idea that alpha frequency promotes temporal processing (for a meta‐analysis, see Samaha & Romei, 2024). While alpha peak frequency normally reaches adult‐like levels during adolescence (Freschl et al., 2022), children with DD show abnormal alpha peak frequency during development (for a review, see Cainelli et al., 2023), suggesting that it might play a role in the pattern of deficits observed in this study. Ultimately, alpha frequency development might not only impact temporal processing itself, but extend to higher‐level abilities impacted in DD, such as phonological encoding (Babiloni et al., 2012).

Impairments in spatiotemporal integration and visual attention have been shown to be related to dysfunctions along the M‐D stream, providing a potential explanation for the neurophysiological alterations ultimately leading to reading impairments in DD (Stein & Walsh, 1997). Since the M‐D stream is the fastest visual subpathway, it is reasonable to believe that its impairment might lead to longer processing times, leading to longer RTs and lower efficiency scores, especially during integration trials in the present study. The temporal resolution of the M‐D stream has been shown to predict RTs in visual perception tasks as well as general reading ability, irrespective of phonological skills or a general performance factor, and is lower in children with DD (McLean et al., 2011). Other studies corroborated this finding by also highlighting relationships between RTs, or other time‐dependent measures in different reading or visual perception tasks, and neurophysiological activity in multiple key structures of the M‐D streams in individuals with DD (Müller‐Axt et al., 2024; Liu et al., 2022; Peters et al., 2020; Kubová et al., 2015; Stein, 2021; Qian & Bi, 2014). Secondly, the deployment of visual attention is slower in DD irrespective of the WM load (Stenneken et al., 2011), and recent computational models predict that the deployment of spatial attention can actually hamper the temporal resolution of the M‐D stream, adding weight to the evidence for the temporal processing impairments of DD (Peñaloza & Ogmen, 2022).

The presence of an integration deficit in children with DD up until ~13 years of age emerging from the present study, together with preserved integration but impaired segregation in adulthood (Ronconi et al., 2022; Santoni et al., 2024), convincingly advocates for complex and non‐linear developmental patterns of integration and segregation abilities. A similar pattern was found by Lallier et al. (2009), where an auditory segregation deficit was present in both children and adults with DD, a visual stream segregation deficit was found in adults but not in children with DD. Developmental factors may be crucial in determining the presence of visual temporal segregation anomalies, such that at later developmental stages the visual system refines its rapid temporal processing capabilities in typical readers, but not in readers with DD (Lallier et al., 2009) who also show a faster degradation of temporal acuity throughout adulthood and beyond (Virsu et al., 2003).

In this regard, while the maturation of the M‐D stream and its connections with other hubs is thought to be complete at ~18 years of age (Langrová et al., 2006), it follows different developmental paths in individuals with DD (Xia et al., 2016) possibly explaining the abnormalities in development. One key unanswered question to understand the differential trajectories of spatiotemporal integration and segregation is whether such delays in the development of integration abilities, which appear compensated in adulthood, might also impact segregation abilities.

Aside from integration and segregation per se, other important insights come from additional group differences. Regardless of task instruction (segregation vs. integration), there was a different sensitivity to the time interval between the two displays of stimuli (i.e., ISI), with a general tendency for participants with DD to worsen their accuracy when the ISI increased (Figure 2b). Notably, the performance pattern of children with DD is similar to the performance of younger children within our sample who showed a decrease in accuracy as the ISI increased (Figure 2d). Moreover, the prediction from our model of inverse efficiency also showed that younger (9.6 y.o.) children with DD became more inefficient as a function of ISI as compared to older children (12.6 y.o.) within the same group (Figure 4c). Relatedly, children with DD showed a stronger improvement in efficiency for long (64 ms) vs. short (16 ms) ISIs.

A possible explanation for these results takes into consideration the developmental course of visuospatial working memory (WM), and potential differences between groups as the ineffective retention of visual units in WM for a sufficiently long period might hinder spatiotemporal integration. Relatedly, recent studies have reported anomalies in the P3 amplitude in populations of children and adults with DD in visual WM tasks (Lotfi et al., 2022; Santoni et al., 2024), which can be attenuated by behavioural training targeting WM (Shiran & Breznitz, 2011) or via transcranial random noise stimulation (tRNS) by plastic modifications in parietal areas (Bertoni et al., 2024) during the attentional blink task, which heavily relies on visual WM (Kranczioch et al., 2005; Ronconi et al., 2016). However, it is important to interpret these results with caution as while analysing the main effect of ISI collapsed across conditions allows to draw inferences on general temporal processing (Freschl et al., 2019; Ronconi et al., 2020; Santoni et al., 2024), condition‐specific effects might still influence the general pattern.

A last important finding was shown when testing the relationship between reading ability and segregation/integration skills in the whole group of participants, with the combined data from the DD and the control group. Correlations showed that performance in both segregation and integration trials in terms of speed and accuracy and inverse efficiency was significantly linked with speed and accuracy measures in standardised reading tests, further suggesting that both integration and segregation mechanisms contribute to the development of efficient reading skills when tested along the continuum of individual reading skills (Figure 5a). Specifically for the integration condition, we found a negative correlation between RTs in integration trials and pseudoword reading speed, indicating that better performance in temporal integration was linked to faster sub‐lexical processing (i.e., faster pseudowords reading). Relatedly, the mean accuracy in integration trials positively correlated with text reading accuracy only in control, but not in DD participants (Figure 5b), suggesting that in typically developing readers, the most ecological reading task (i.e., text reading) relies on the correct development of temporal integration mechanisms. Such integration presumably allows information units (e.g., graphemes, syllables) to be efficiently coded in order to find a proper lexical match.

It is important to recognise a limitation concerning the sample of the present study. We could not map all the ages until adulthood, where different patterns emerge (Ronconi et al., 2022; Santoni et al., 2024). Considering that in some of our model integration and segregation deficits should fade when reaching ~13 years of age (Figure 4a), it is very reasonable to assume that the point in time where possible non‐linearities or trend inversion emerge was not sampled. Therefore, to fully understand the developmental pattern of integration segregation abilities, it might be necessary to develop further studies with higher statistical power, sampling a larger age span in a stratified way.

In conclusion, we presented novel evidence showing the developmental trajectory of spatiotemporal processing deficits observed in DD. Our findings revealed differentiated developmental trajectories of integration and segregation abilities possibly stemming from several causes including the concurrent development of the M‐D stream and alpha oscillations in line with visuo‐attentional theories of DD, while also highlighting the role of visual WM. Future studies should possibly jointly investigate all of these aspects across development and ageing by also including neuroimaging techniques to clarify their individual roles in spatiotemporal processing.

## AUTHOR CONTRIBUTIONS

**Giuseppe Di Dona:** Methodology; software; formal analysis; visualisation; data curation; writing – original draft; writing – review and editing. **Alessia Santoni:** Methodology; investigation; data curation; writing – original draft; writing – review and editing. **David Melcher:** Conceptualisation; methodology; software; writing – review and editing. **Luca Ronconi:** Conceptualisation; methodology; software; writing – original draft; writing – review and editing; supervision. **Laura Franchin:** Conceptualisation; methodology; software; resources; investigation; writing – review and editing; supervision; project administration.

## CONFLICT OF INTEREST STATEMENT

The authors declare no conflicts of interest.

## Supporting information


Data S1:

## Data Availability

The data that support the findings of this study are not publicly available due to ethical restrictions.

## Footnotes

^1^
Data collection occurred in a country where data on race/ethnicity cannot legally be collected.
