# Supplementary material for: Developmental trajectories of visual temporal integration and segregation in children with and without developmental dyslexia
Source: Br J Dev Psychol. 2025 Aug 21;44(1):146–65. doi: 10.1111/bjdp.70010 (PMC12884368; doi:10.1111/bjdp.70010)
Supplement: Supplementary file 1 — Data S1: [file BJDP-44-146-s001.docx]

**Supplementary Materials**

1. **Supplementary Table 1: Demographics and Comorbidities of the Developmental Dyslexia Group**

| **ID** | **Age** | **Sex** | **Dyslexia** | **Dysorthography** | **Dyscalculia** | **Dysgraphia** |
| --- | --- | --- | --- | --- | --- | --- |
| 101 | 10 | M | ✓ | ✓ |  |  |
| 102 | 11 | F | ✓ | ✓ | ✓ | ✓ |
| 103 | 10 | M | ✓ | ✓ | ✓ | ✓ |
| 104 | 12 | M | ✓ | ✓ | ✓ | ✓ |
| 105 | 10 | M | ✓ | ✓ |  |  |
| 106 | 13 | M | ✓ | ✓ |  |  |
| 107 | 14 | M | ✓ | ✓ | ✓ | ✓ |
| 108 | 12 | M | ✓ |  | ✓ |  |
| 110 | 12 | F | ✓ |  |  |  |
| 111 | 12 | M | ✓ | ✓ |  | ✓ |
| 112 | 9 | M | ✓ | ✓ | ✓ | ✓ |
| 113 | 10 | F | ✓ | ✓ |  |  |
| 119 | 12 | M | ✓ |  |  | ✓ |
| 120 | 11 | M | ✓ | ✓ | ✓ | ✓ |
| 121 | 14 | F | ✓ | ✓ | ✓ |  |
| 122 | 10 | M | ✓ |  |  |  |
| 123 | 12 | M | ✓ | ✓ |  |  |
| 124 | 10 | M | ✓ | ✓ |  |  |

1. **Supplementary Table 2**

| **Developmental Dyslexia (N = 19)** | | | | | | | | | | |  | |  | |  |
| --- | --- | --- | --- | --- | --- | --- | --- | --- | --- | --- | --- | --- | --- | --- | --- |
|  | ***Mean Accuracy Integration*** | ***Mean Accuracy Segregation*** | ***Mean RTs Integration*** | ***Mean RTs Segregation*** | ***Raven Score*** | ***Text Speed*** | ***Text Accuracy*** | ***Words Speed*** | ***Words Accuracy*** | ***Pseudowords Speed*** | | ***Pseudowords Accuracy*** | | ***Mean Inveff Integration*** |  |
| ***Mean Inveff Segregation*** | -0.35 | -0.86*** | 0.56* | 0.58* | 0.28 | -0.06 | 0.43 | -0.13 | 0.36 | 0.07 | 0.33 | | 0.42 | |  |
| ***Mean Inveff Integration*** | -0.68** | -0.59** | 0.82*** | 0.67** | 0.05 | -0.03 | -0.16 | -0.07 | 0.01 | 0.24 | 0.11 | |  | |  |
| ***Pseudowords Accuracy*** | 0.08 | -0.15 | 0.41 | 0.53* | -0.16 | 0.1 | 0.74*** | -0.07 | 0.74*** | -0.17 |  | |  | |  |
| ***Pseudowords Speed*** | 0.18 | -0.26 | 0.04 | 0.02 | -0.27 | 0.18 | -0.05 | 0.42 | 0.04 |  |  | |  | |  |
| ***Words Accuracy*** | 0.07 | -0.24 | 0.32 | 0.45 | -0.07 | -0.18 | 0.6** | 0.02 |  |  |  | |  | |  |
| ***Words Speed*** | 0.11 | 0.04 | -0.23 | -0.22 | -0.36 | 0.43 | -0.01 |  |  |  |  | |  | |  |
| ***Text Accuracy*** | 0.23 | -0.23 | 0.01 | 0.24 | -0.17 | 0.03 |  |  |  |  |  | |  | |  |
| ***Text Speed*** | 0.25 | -0.01 | -0.12 | -0.06 | -0.06 |  |  |  |  |  |  | |  | |  |
| ***Raven Score*** | 0.03 | -0.02 | 0.18 | 0.09 |  |  |  |  |  |  |  | |  | |  |
| ***Mean RTs Segregation*** | -0.43 | -0.66** | 0.88*** |  |  |  |  |  |  |  |  | |  | |  |
| ***Mean RTs Integration*** | -0.56* | -0.59** |  |  |  |  |  |  |  |  |  | |  | |  |
| ***Mean Accuracy Segregation*** | 0.53* |  |  |  |  |  |  |  |  |  |  | |  | |  |
| **Control (N = 24)** | | | | | | | | | | |  | |  | |  |
|  | ***Mean Accuracy Integration*** | ***Mean Accuracy Segregation*** | ***Mean RTs Integration*** | ***Mean RTs Segregation*** | ***Raven Score*** | ***Text Speed*** | ***Text Accuracy*** | ***Words Speed*** | ***Words Accuracy*** | ***Pseudowords Speed*** | ***Pseudowords Accuracy*** | | ***Mean Inveff Integration*** | |  |
| ***Mean Inveff Segregation*** | -0.1 | -0.73*** | 0.17 | 0.54 | -0.17 | -0.04 | -0.21 | -0.07 | -0.08 | 0.18 | -0.04 | | 0.11 | |  |
| ***Mean Inveff Integration*** | -0.59*** | 0.23 | 0.78*** | 0.47* | 0.06 | -0.02 | -0.25 | -0.06 | -0.04 | -0.01 | -0.1 | |  | |  |
| ***Pseudowords Accuracy*** | 0.27 | 0.17 | 0.07 | 0.13 | 0.46* | 0.32 | 0.7*** | 0.28 | 0.76*** | 0.08 |  | |  | |  |
| ***Pseudowords Speed*** | -0.13 | -0.08 | 0 | 0.13 | 0.16 | 0.65*** | -0.13 | 0.76*** | 0.18 |  |  | |  | |  |
| ***Words Accuracy*** | 0.22 | 0.28 | 0.08 | 0.04 | 0.38 | 0.61*** | 0.53 | 0.54 |  |  |  | |  | |  |
| ***Words Speed*** | -0.08 | 0.25 | -0.04 | 0.08 | 0.4 | 0.8*** | 0.03 |  |  |  |  | |  | |  |
| ***Text Accuracy*** | 0.41* | 0.19 | -0.09 | -0.31 | 0.22 | -0.01 |  |  |  |  |  | |  | |  |
| ***Text Speed*** | 0.08 | 0.15 | -0.07 | 0 | 0.33 |  |  |  |  |  |  | |  | |  |
| ***Raven Score*** | 0.05 | 0.43* | 0.14 | 0.15 |  |  |  |  |  |  |  | |  | |  |
| ***Mean RTs Segregation*** | -0.18 | -0.24 | 0.73*** |  |  |  |  |  |  |  |  | |  | |  |
| ***Mean RTs Integration*** | -0.27 | 0.22 |  |  |  |  |  |  |  |  |  | |  | |  |
| ***Mean Accuracy Segregation*** | -0.18 |  |  |  |  |  |  |  |  |  |  | |  | |  |
| **All participants (N = 43)** | | | | | | | | | | |  | |  | |  |
|  | ***Mean Accuracy Integration*** | ***Mean Accuracy Segregation*** | ***Mean RTs Integration*** | ***Mean RTs Segregation*** | ***Raven Score*** | ***Text Speed*** | ***Text Accuracy*** | ***Words Speed*** | ***Words Accuracy*** | ***Pseudowords Speed*** | ***Pseudowords Accuracy*** | | ***Mean Inveff Integration*** | |  |
| ***Mean Inveff Segregation*** | -0.24 | -0.84*** | 0.64*** | 0.66*** | 0.01 | -0.34* | -0.01 | -0.42** | -0.01 | -0.31* | 0 | | 0.59*** | |  |
| ***Mean Inveff Integration*** | -0.55*** | -0.6*** | 0.86*** | 0.72*** | -0.1 | -0.45** | -0.46** | -0.53*** | -0.3 | -0.37* | -0.27 | |  | |  |
| ***Pseudowords Accuracy*** | 0.37* | 0.19 | -0.03 | 0.09 | 0.2 | 0.55*** | 0.85*** | 0.49** | 0.79*** | 0.36* |  | |  | |  |
| ***Pseudowords Speed*** | 0.28 | 0.24 | -0.36* | -0.31* | 0.11 | 0.68*** | 0.46** | 0.79*** | 0.41** |  |  | |  | |  |
| ***Words Accuracy*** | 0.3 | 0.18 | -0.06 | 0.05 | 0.24 | 0.44** | 0.7*** | 0.49** |  |  |  | |  | |  |
| ***Words Speed*** | 0.32* | 0.44** | -0.51*** | -0.44** | 0.16 | 0.81*** | 0.56*** |  |  |  |  | |  | |  |
| ***Text Accuracy*** | 0.46** | 0.21 | -0.31* | -0.17 | 0.14 | 0.55*** |  |  |  |  |  | |  | |  |
| ***Text Speed*** | 0.39* | 0.37* | -0.42** | -0.34* | 0.26 |  |  |  |  |  |  | |  | |  |
| ***Raven Score*** | 0.12 | 0.26 | 0.01 | -0.01 |  |  |  |  |  |  |  | |  | |  |
| ***Mean RTs Segregation*** | -0.35* | -0.67*** | 0.89*** |  |  |  |  |  |  |  |  | |  | |  |
| ***Mean RTs Integration*** | -0.44** | -0.57*** |  |  |  |  |  |  |  |  |  | |  | |  |
| ***Mean Accuracy Segregation*** | 0.27 |  |  |  |  |  |  |  |  |  |  | |  | |  |

1. **Supplementary Figure 1**

**
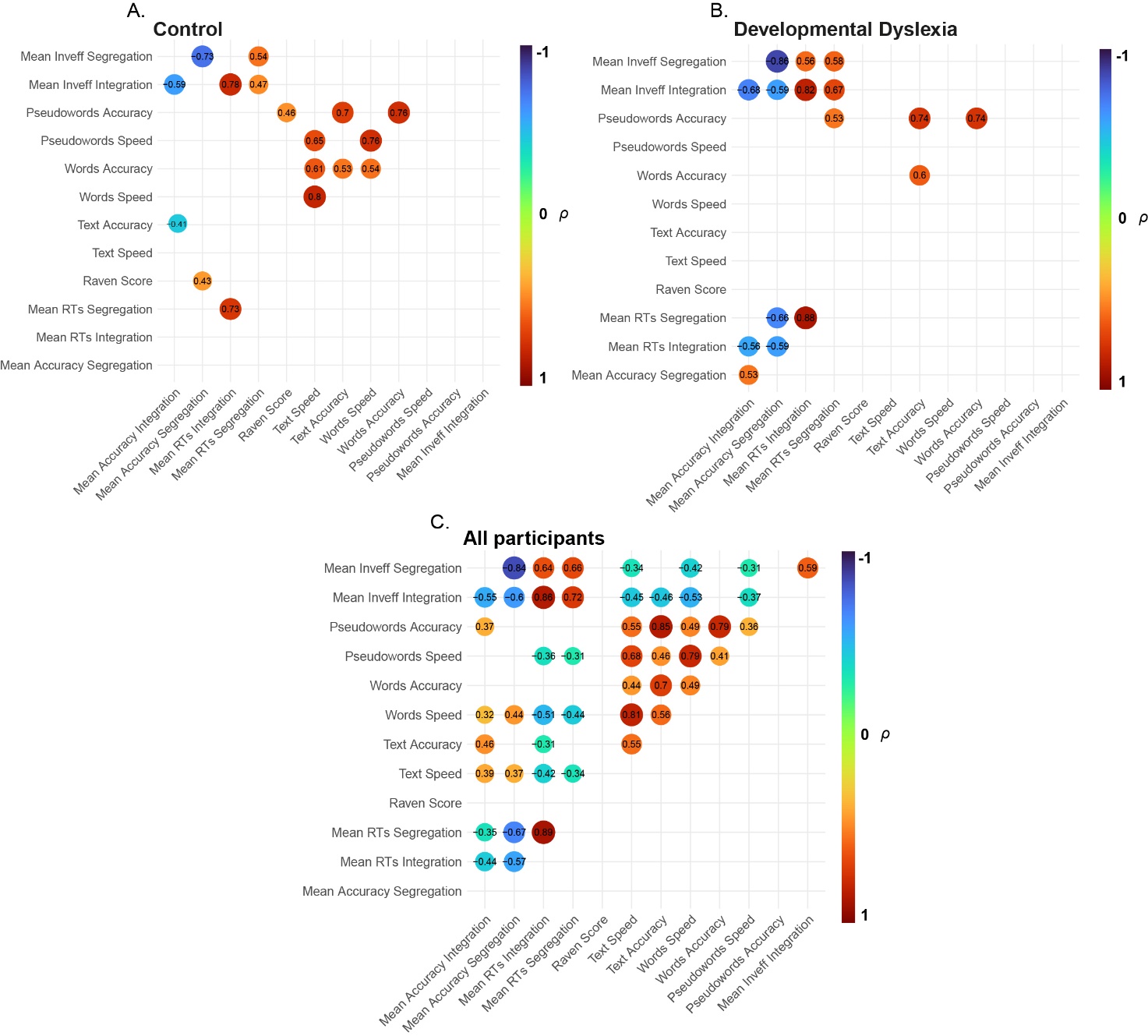
**
